# Impact on survival of whole-body computed tomography before emergency bleeding control in patients with severe blunt trauma

**DOI:** 10.1186/cc12861

**Published:** 2013-08-27

**Authors:** Daiki Wada, Yasushi Nakamori, Kazuma Yamakawa, Yoshiaki Yoshikawa, Takeyuki Kiguchi, Osamu Tasaki, Hiroshi Ogura, Yasuyuki Kuwagata, Takeshi Shimazu, Toshimitsu Hamasaki, Satoshi Fujimi

**Affiliations:** 1 Department of Emergency and Critical Care, Osaka General Medical Center, 3-1-56 Bandai-Higashi, Sumiyoshi-ku, Osaka 558-8558, Japan; 2 Department of Emergency and Critical Care Medicine, Kansai Medical University Hirakata Hospital, 2-3-1 Shinmachi, Hirakata, Osaka 573-1191, Japan; 3 Department of Traumatology and Acute Critical Medicine, Osaka University Graduate School of Medicine, 2-15 Yamadaoka, Suita, Osaka 565-0871, Japan; 4 Department of Emergency Medicine, Unit of Clinical Medicine, Nagasaki University Graduate School of Biomedical Sciences, 1-7-1 Sakamoto, Nagasaki 852-8501, Japan; 5 Department of Biomedical Statistics, Osaka University Graduate School of Medicine, 2-15 Yamadaoka, Suita, Osaka 565-0871, Japan

## Abstract

**Introduction:**

Whole-body computed tomography (CT) has gained importance in the early diagnostic phase of trauma care. However, the diagnostic value of CT for seriously injured patients is not thoroughly clarified. This study assessed whether preoperative CT beneficially affected survival of patients with blunt trauma who required emergency bleeding control.

**Methods:**

This retrospective study was conducted from January 2004 to December 2010 in two tertiary trauma centers in Japan. The primary inclusion criterion was patients with blunt trauma who required emergency bleeding control (surgery or transcatheter arterial embolization). CT before emergency bleeding control was performed at the attending physician's discretion based on individual patient condition (for example, hemodynamic stability or certain abnormalities in the primary survey). We assessed covariates associated with 28-day mortality with multivariate logistic regression analysis and evaluated standardized mortality ratio (SMR, ratio of observed to predicted mortality by Trauma and Injury Severity Score (TRISS) method) in two subgroups of patients who did or did not undergo CT.

**Results:**

The inclusion criterion was fulfilled by 152 patients with a median Injury Severity Score of 35.3. During the early resuscitation phase, 132 (87%) patients underwent CT and 20 (13%) did not. Severity of injury was significantly higher in the non-CT versus CT group patients. Observed mortality rate was significantly lower in the CT versus non-CT group (18% vs. 80%, *P *<0.001). Multivariate adjustment for the probability of survival (Ps) by TRISS method confirmed CT as an independent predictor for 28-day mortality (adjusted OR, 7.22; 95% CI, 1.76 to 29.60; *P *= 0.006). In the subgroup with less severe trauma (TRISS Ps ≥50%), SMR in the CT group was 0.63 (95% CI, 0.23 to 1.03; *P *= 0.066), indicating no significant difference between observed and predicted mortality in the CT group. In contrast, in the subgroup with more severe trauma (TRISS Ps <50%), SMR was 0.65 (95% CI, 0.41 to 0.90; *P *= 0.004) only in the CT group, whereas the difference between observed and predicted mortality was not significant in the non-CT group, suggesting a possible beneficial effect of CT on survival only in trauma patients at high risk of death.

**Conclusion:**

CT performed before emergency bleeding control might be associated with improved survival, especially in severe trauma patients with TRISS Ps of <50%.

## Introduction

Approximately 24,000 trauma deaths occur in Japan annually, making trauma the fifth leading cause of death, and among young adults, trauma is the leading cause of death [1]. Preventable trauma deaths are defined as those where significant delays occur before exsanguinating hemorrhage is controlled [2]. Sufficient therapy within the first hour after trauma significantly increases the patient's chance for survival. Thus, therapeutic procedures and diagnostic evaluation have to be concomitant events performed by a multidisciplinary team [3]. To decrease preventable trauma deaths, the Advanced Trauma Life Support (ATLS) concept has gained wide acceptance as a standardized way to systematically manage the trauma patient by a team [4,5]. In Japan, the Japan Advanced Trauma Evaluation and Care (JATEC) program based on the ATLS concept has gained wide acceptance [1].

To improve the workflow of acute trauma care, the management of severely injured patients is a matter of ongoing development. In particular, among imaging modalities, whole-body computed tomography (CT) represents a substantial refinement in the diagnostic work-up of multitrauma patients [6]. The process quality of whole-body CT has been proven in several studies that have confirmed its feasibility, high diagnostic safety, and substantial reduction in scan time [7]. Salim *et al. *showed that whole-body CT resulted in a change of treatment in 19% of 1,000 patients without obvious external signs of injuries [8]. CT has gained importance in the early diagnostic phase of trauma care and has become an essential part of the trauma diagnostic work-up [9]. Hilbert *et al. *reported that by integrating CT into the trauma room, elimination of patient transfer from the emergency room to the CT scanner location is of enormous benefit in terms of work-up time [10]. However, few studies have examined the benefit of whole-body CT on mortality in patients with severe blunt trauma who required emergency bleeding control. Therefore, the objective of this study was to assess whether preoperative whole-body CT had a beneficial impact on survival in blunt trauma patients who required emergency bleeding control.

## Materials and methods

### Patient population

**Figure 1:**
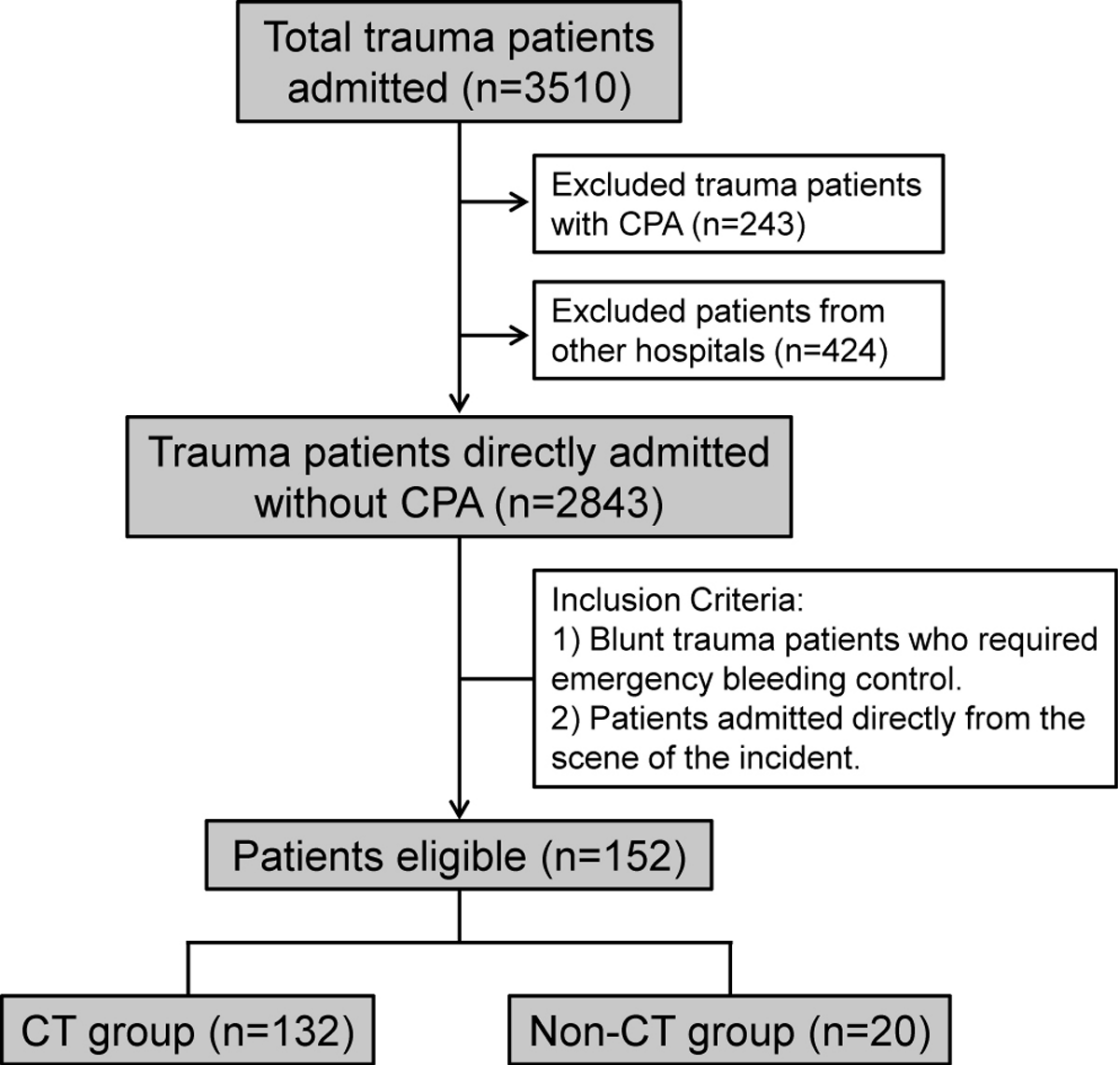
**Patient flow diagram**. CPA, cardiopulmonary arrest.

This was a retrospective study conducted from January 2004 to December 2010 at the Osaka General Medical Center and Osaka University Graduate School of Medicine. Inclusion criteria were as follows: patients with blunt trauma, who required emergency bleeding control and, who were admitted directly from the scene of the incident. Emergency bleeding control was defined as any emergent thoracotomy, laparotomy or transcatheter arterial embolization procedure performed for control of chest, abdominal or pelvic organ bleeding in the emergency department or operating room. Exclusion criteria included patients who were transferred from other hospitals; patients with traumatic cardiopulmonary arrest on arrival; and patients with severe brain injury that was the direct cause of death. The patient flow diagram is shown in Figure 1.

This study followed the principles of the Declaration of Helsinki, and the conduction of this study was approved by the institutional review board at Osaka General Medical Center. The board waived the need for informed consent because this was a retrospective chart review study.

### Trauma management policy

The two institutions participating in the present study are tertiary referral hospitals where together, over 700 trauma patients are admitted annually. Both hospitals chose the same diagnostic algorithm, that of the JATEC program, which is based on the ATLS concept [4]. Briefly, in the primary survey of the patient, focused assessment with sonography for trauma (FAST) and chest and pelvic X-ray examinations are performed for diagnosis. If available, CT is performed before emergency bleeding control. Each physician decides not to perform CT if certain abnormalities are clearly identified in the FAST and X-ray images or if patient transfer is difficult due to hemodynamic instability. The locations of the CT scanners in both institutions are on the same floor as the trauma rooms. The time required to perform the CT scan, which includes patient transfer time, is about 20 minutes.

### Data collection

Patients were followed until 28 days after admission. Emergency department variables (systolic blood pressure (SBP), heart rate, respiratory rate, shock index (SI), base excess (BE) and lactate value) were recorded as the initial set of vital signs. Body temperature (BT) and prothrombin time (PT) recorded were those with the lowest level within 24 hours from the time of arrival at the hospital. In-hospital variables (fluid infusion, blood transfusion, fresh frozen plasma (FFP) transfusion) were recorded as the total volume of each within 24 hours. We then calculated Injury Severity Score (ISS), Revised Trauma Score (RTS), and the probability of survival (Ps) by the Trauma and Injury Severity Score (TRISS) method. TRISS method is the most widely used method for measurement of expected outcome in patients with trauma [11-14]. The primary outcome event for analysis was 28-day mortality.

### Statistical analysis

Data are expressed as group medians with interquartile ranges or numbers with percentages, as appropriate. Continuous variables were compared between groups with the Mann-Whitney *U *test. Categorical variables were analyzed with the *χ*^2 ^test or Fisher's exact test, as appropriate.

Multivariate regression analysis was used to assess the covariates that were associated with 28-day mortality. We then performed an outcome analysis to calculate standardized mortality ratio (SMR; ratio of observed to predicted mortality calculated by TRISS method). Observed mortality was compared with predicted mortality with the Wald-type test with logistic regression. We divided all of the patients into two groups based on whether TRISS Ps was ≥50% or <50% to assess whether CT before emergency bleeding control improved survival especially in patients at high risk of death (TRISS Ps <50%). In addition, the CT group patients were divided into two subgroups based on whether SI just before they underwent CT was ≥1 or <1 to assess whether CT before emergency bleeding control improved survival, especially in hemodynamically unstable patients (SI ≥1).

A *P-*value of <0.05 was considered to indicate statistical significance. Statistical analyses were performed with SPSS for Windows version 17.0 software (SPSS, Inc., Chicago, IL, USA) and SAS Statistical Software version 9.1.3 (SAS Institute Inc., Cary, NC, USA).

## Results

### Baseline characteristics

Baseline characteristics of the 152 patients who met the entry criteria are shown in Table 1. This study cohort (median age, 40 (25 to 61) years) represented a significantly injured population with a median ISS of 35.3 and an overall 28-day mortality of 26.3%. Of these 152 patients, 132 underwent CT imaging before emergency surgical bleeding control (CT group) and 20 did not (non-CT group). The TRISS method could be applied to all 152 patients. As an indication of the severity of trauma, the ISS, SBP, RTS, BE and lactate levels, number of blood transfusions within 24 hours, number of FFP transfusions within 24 hours, and TRISS Ps were all significantly lower in the non-CT group compared with the CT group. The median time from patient arrival to the start of emergency bleeding control in the non-CT group was 74 (63 to 114) minutes, significantly shorter than that of 84 (67 to 121) minutes in the CT group. The 28-day mortality rate was also significantly higher in the non-CT group versus the CT group (80% vs. 18%, *P *<0.001).

**Table 1:** Baseline characteristics and diagnostic data of the study population

	Total (*n *= 152)	CT group(*n *= 132)	Non-CT group(*n *= 20)	*P-*value
**Baseline characteristics**				
Age, yrs	40 (25 to 61)	40 (26 to 61)	42 (23 to 64)	n.s.
Male sex, %	99 (65.1)	86 (65)	13 (65)	n.s.
**Emergency department variables**				
Systolic blood pressure, mmHg	93.5 (70 to 122)	99 (80 to 128)	62 (50 to 74)	<0.001
Heart rate, bpm	101 (83 to 124)	98 (82 to 122)	105 (100 to 132)	n.s.
Respiratory rate, bpm	23 (20 to 30)	23 (20 to 30)	20 (20 to 28)	n.s.
Revised Trauma Score	6.9 (5.3 to 7.8)	7.0 (6.3 to 7.8)	4.3 (3.2 to 5.6)	<0.001
Base excess, mmol/L	-5.0 (-8 to -1.8)	-4.0 (-7.6 to -1.4)	-9.7 (-13 to -7.4)	<0.001
Lactate, mmol/L	3.9 (2.5 to 7.0)	3.5 (2.4 to 6.0)	8.1 (6.0 to 8.8)	<0.001
**In-hospital variables**				
Body temperature, °C	35.2 (34.5 to 35.8)	35.2 (34.5 to 35.8)	34.7 (34 to 35.6)	n.s.
Prothrombin time, %	50 (34.2 to 65)	50 (35.5 to 67.2)	40 (32.7 to 60.2)	n.s.
Infusion within 24 hrs, ml	11,000 (8,625 to 16,000)	14,000 (9,000 to 16,000)	10,500 (7,750 to 15,250)	n.s.
Blood transfusion within 24 hrs, units	14 (6 to 25.5)	18 (9.0 to 31)	23 (16 to 44)	0.001
FFP transfusion within 24 hrs, units	3.0 (0 to 10)	0.0 (0 to 10)	8.0 (3.0 to 13)	0.045
**Bleeding control site**				
Chest	21 (13.8)	15 (11.3)	6 (30)	n.s.
Abdomen	93 (61.1)	78 (59)	15 (75)	n.s.
Pelvic	70 (46)	60 (45.4)	10 (50)	n.s.
**Time from patient arrival to start of emergency bleeding control, minutes**	79 (55 to 105)	84 (67 to 121)	74 (63 to 114)	<0.001
**Injury Severity Score**	34 (25 to 45)	34 (25 to 43)	41 (34 to 51)	0.033
**TRISS Ps, %**	80 (39.1 to 95.1)	84.5 (56.5 to 95.5)	22.2 (7.9 to 39.2)	<0.001
**Outcome variables**				
24-hr mortality, %	39 (25.6)	23 (17.4)	16 (80)	<0.001
28-d mortality, %	40 (26.3)	24 (18.1)	16 (80)	<0.001

Data are expressed as group medians with interquartile ranges or number (percent). bpm, beats per minute; CT, computed tomography; FFP, fresh frozen plasma; n.s., not significant; Ps, probability of survival; TRISS, Trauma and Injury Severity Score.

### Effect of CT on mortality by multivariate logistic regression analysis

Because significant differences existed in baseline severity of trauma between the two CT groups, multivariate logistic regression analysis was applied to adjust for possible confounders. Covariates to estimate the effect of CT in the regression model were TRISS Ps in model 1, and BE, BT and PT in model 2, as shown in Table 2. Consequently, CT was found to be an independent predictor for survival that added significant predictive power to both models (model 1: odds ratio (OR), 7.224; 95% confidential interval (CI), 1.763 to 29.601; *P *= 0.006 and model 2: OR, 11.745; 95% CI, 3.313 to 41.637; *P *<0.001).

**Table 2:** Results of multivariate logistic regression analysis

Covariate	Coeff(β)	SE(β)	OR	95% CI	*P-*value
**Model 1: (CT+TRISS Ps)**					
CT	1.977	0.720	7.224	1.763 to 29.601	0.006
TRISS Ps	0.044	0.008	1.045	1.029 to 1.061	<0.001
**Model 2: (CT+BE+BT+PT)**					
CT	2.463	0.646	11.745	3.313 to 41.637	<0.001
BE	0.128	0.047	1.137	1.036 to 1.247	0.007
BT	0.444	0.202	1.559	1.050 to 2.316	0.028
PT	0.015	0.012	1.015	0.992 to 1.038	0.198

BE, base excess; BT, body temperature; CI, confidence interval; Coeff(β), coefficient; CT, computed tomography; OR, odds ratio; Ps, probability of survival; PT, prothrombin time; SE(β), standard error of coefficient; TRISS, Trauma and Injury Severity Score.

### Effect of CT on mortality by standard mortality ratio analysis

**Figure 2:**
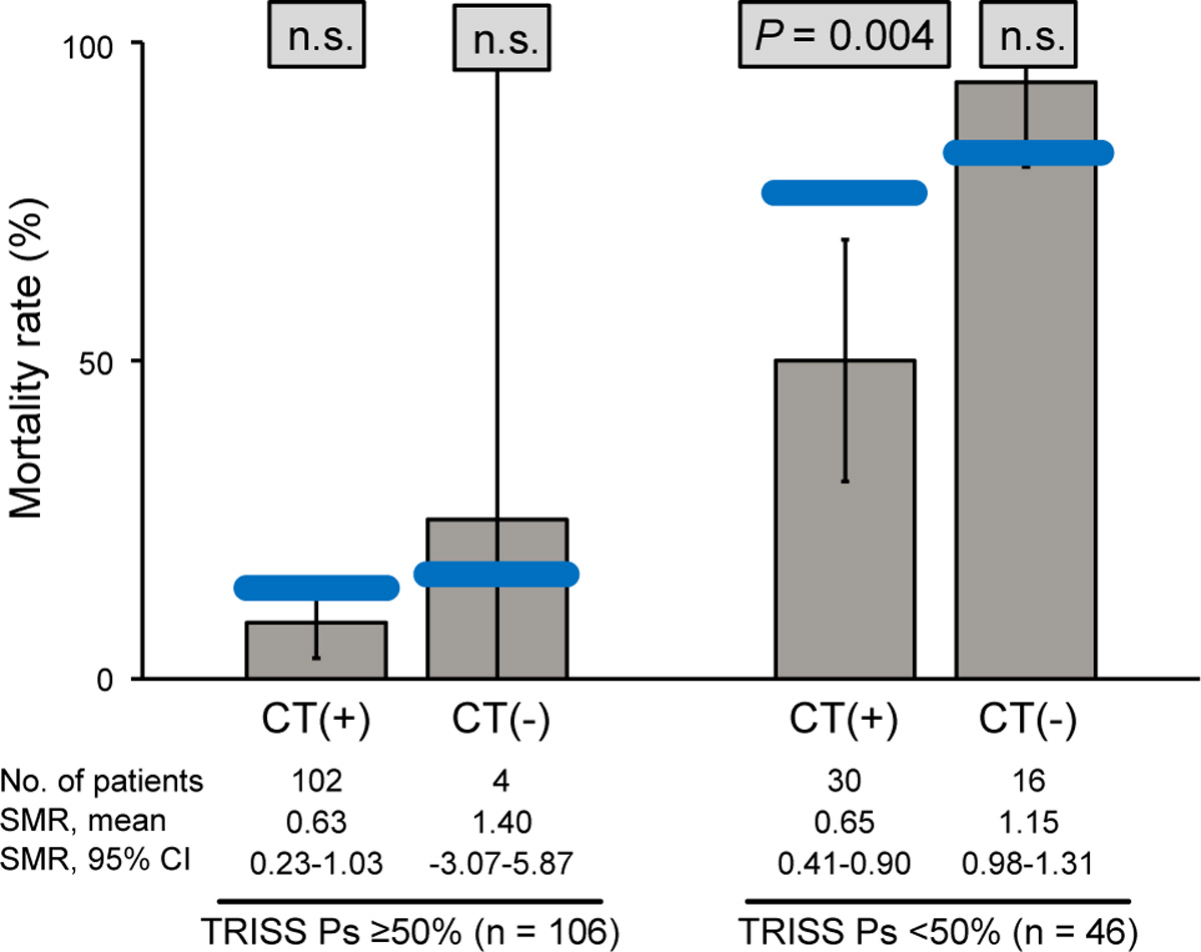
**Outcome analysis for calculation of standardized mortality ratio (SMR) on the basis of the Trauma and Injury Severity Score (TRISS) method**. All patients were divided into two groups on the basis of TRISS Ps. The gray columns represent observed mortality rates, the blue bars represent predicted mortality rates, and the whisker bars represent the 95% confidence range. Ps, probability of survival

In the subgroup with less severe trauma (TRISS Ps ≥50%), SMR showed no significant difference between observed and predicted mortality either in the CT group or the non-CT group (Figure 2). In the subgroup with more severe trauma (TRISS Ps <50%), when comparing observed mortality with predicted mortality, results in the CT group showed observed mortality of 50% versus predicted mortality of 76.4%. Thus, SMR showed a significant difference only in the CT group (SMR, 0.65; 95% CI, 0.41 to 0.9; *P *= 0.004), indicating that observed mortality was significantly lower than predicted mortality, whereas this was not the case in the non-CT group.

**Figure 3:**
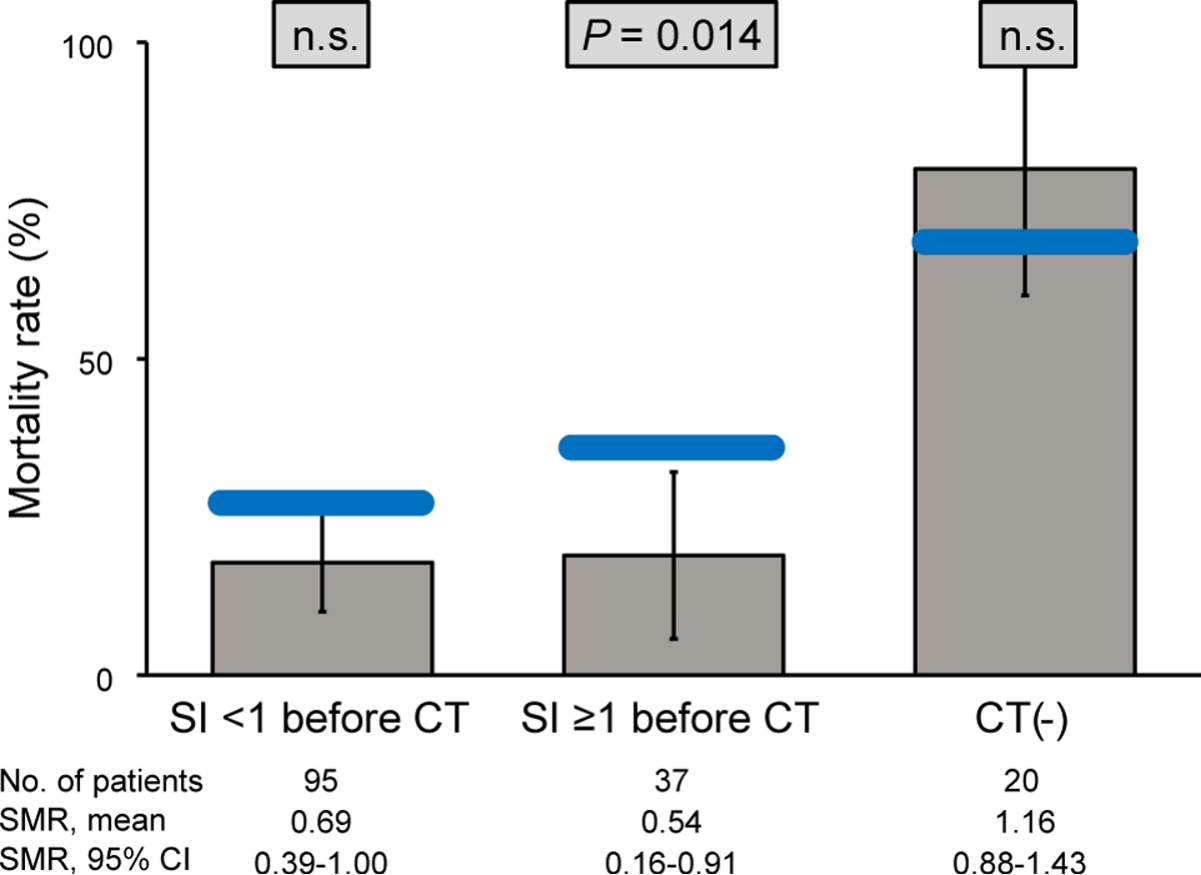
**Outcome analysis for calculation of standardized mortality ratio (SMR) on the basis of shock index (SI) value**. The patients who underwent CT scanning were divided into two groups on the basis of their SI value. The gray columns represent observed mortality rates, the blue bars represent predicted mortality rates, and the whisker bars represent the 95% confidence range.

In addition, in the hemodynamically unstable subgroup (SI just before CT of ≥1), SMR showed a significant difference only in the CT group (SMR, 0.54; 95% CI, 0.16 to 0.91; *P *= 0.014) (Figure 3), indicating that observed mortality was significantly lower than predicted mortality. In the hemodynamically stable subgroup (SI just before CT <1), SMR showed no significant difference in the CT group.

### Effect of CT in the patients with multiple injuries

The number of patients requiring emergency bleeding control in more than one body region (chest, abdomen and pelvic) was 21 (15.9%) in the CT group and 10 (50%) in the non-CT group. In the CT group, 14 of 21 (66.6%) patients lived, whereas only 2 of 10 (20%) patients lived in the non-CT group (*P *= 0.015). In the patients with a TRISS Ps of <50%, the number of patients requiring emergency bleeding control in more than one body region was nine (30%) in the CT group and seven (43.8%) in the non-CT group. In the CT group, four of nine (44.4%) patients lived, whereas in the non-CT group, none of the seven (0%) patients lived (*P *= 0.042).

## Discussion

Multivariate analysis revealed CT to be an independent predictor for probability of 28-day survival in patients with severe blunt trauma who required emergency bleeding control. In addition, in the subgroup with more severe trauma (TRISS Ps <50%) and the hemodynamically unstable subgroup (SI just before CT of ≥1), we observed a better survival rate for CT patients than that predicted by TRISS method, whereas there was no difference in survival rate of non-CT patients. The results of this study provide the first evidence, to our knowledge, that CT offers a significant beneficial effect on mortality in the early management of severe blunt trauma.

The ATLS guidelines clearly state that after a quick "first survey", resuscitation of the patient has priority over advanced diagnostic procedures. When the patient is hemodynamically unstable, the patient is usually examined clinically and undergoes diagnostic procedures (conventional radiography and FAST) and CT scanning after emergency surgery [4]. Clarke *et al. *reported that delay to laparotomy in patients with intra-abdominal hemorrhage after trauma was associated with an increased risk of mortality [15]. Neal *et al. *reported that delay secondary to abdominal CT in patients who require operative intervention results in an independent higher risk of mortality [16].

Improvements in technology have brought about a change in the use of CT in trauma treatment. Recent technological advances related to the introduction of CT have led to increasing use of whole-body CT thanks to a reduction in data acquisition time and improvement in the quality of imaging data [17]. Ptak *et al. *could show that whole-body multidetector CT shortens scan time compared with that of single-detector helical CT, from 41 to 3 minutes, and patient throughput time from 65 to 23 minutes [18]. Huber-Wagner *et al. *reported that integration of whole-body CT into early trauma care significantly increases the probability of survival in patients with polytrauma using the data recorded in the trauma registry of the German Trauma Society [19]. Wurmb *et al. *reported that rapid diagnostic workup with whole-body CT might be associated with an improved outcome if emergency surgery is necessary for seriously injured patients [20]. However, the importance of this technology in early trauma management remains controversial. Still, little is known about the diagnostic value and the therapeutic impact on mortality when preoperative CT is performed for blunt trauma patients requiring emergency bleeding control, especially for patients at high risk of death and for hemodynamically unstable patients.

The present study revealed that preoperative CT was associated with improved survival in patients at high risk of death with a low TRISS Ps and in hemodynamically unstable patients, whereas CT did not improve survival in the patients with less severe trauma or who were hemodynamically stable. There are two possibilities for why CT improved survival in the patients with a TRISS Ps of <50%. First, these patients actually require emergency bleeding control in more than one body region, such as the thoracic, abdominal and pelvic regions. CT helps in the decision to prioritize the type of emergency bleeding control required on the first approach and was helpful in promptly tailoring subsequent treatment. In the patients in the present study with more than one site of bleeding, survival rate in the CT group was significantly higher than that in the non-CT group. Fang *et al. *reported that the priority of emergency surgery or angiography can be individualized and customized according to the CT findings [21]. Second, unexpected sites of bleeding were more often discovered during emergency bleeding control in the non-CT group than in the CT group. CT performed before emergency bleeding control contributed to the avoidance of a high rate of unexpected bleeding. Neal *et al. *reported that abdominal CT would result in delay and a greater risk of mortality after significant abdominal injury [16]. However, they focused on a trauma population with isolated abdominal injuries requiring laparotomy so that there might be few patients with a TRISS Ps of <50% in their study. Further clinical investigation is necessary to clarify in which population with severe trauma CT will have the most significant effect on patient outcome.

We acknowledge several limitations of an observational study design. This is a retrospective observational study and not a randomized control study. The study was conducted in only two institutions, and the sample size was small in the non-CT group (*n *= 20). Significant differences in baseline severity of trauma, as indicated by the TRISS Ps and ISS score, existed between the two groups. In addition, the decision to perform CT may have introduced major selection bias in the present study because CT was performed at the discretion of the attending physician based on individual patient condition and not according to a pre-defined protocol. The combination of these limitations might cause multiple unmeasured variables to account for the outcome differences observed in this study. Considering these possible confounders, we performed multivariate logistic regression analysis and SMR analysis.

## Conclusion

Whole-body CT performed before emergency bleeding control might be associated with improved survival, especially in patients at high risk of death and in hemodynamically unstable patients.

## Key messages

• CT was an independent predictor for the probability of 28-day survival in patients with severe blunt trauma who required emergency bleeding control.

• CT was associated with 28-day survival, especially in patients at high risk of death (TRISS Ps <50%) and in the hemodynamically unstable subgroup (shock index calculated just before CT of >1).

## Abbreviations

ATLS: Advanced Trauma Life Support; BE: base excess; BT: body temperature; CI: confidential interval; CT: computed tomography; FAST: focused assessment with sonography for trauma; FFP: fresh frozen plasma; ISS: Injury Severity Score; JATEC: Japan Advanced Trauma Evaluation and Care; OR: odds ratio; Ps: probability of survival; PT: prothrombin time; RTS: Revised Trauma Score; SBP: systolic blood pressure; SI: shock index; SMR: standardized mortality ratio; TRISS: Trauma and Injury Severity Score.

## Competing interests

The authors declare that they have no competing interests.

## Authors' contributions

DW participated in the study design and in data collection and interpretation, and drafted the manuscript. YN conceived the study and its design and helped to draft the manuscript. YY and TK participated in data interpretation. HO, YK, TS and SF participated in study design and data collection. KY and OT had a major impact on the interpretation of data and critical appraisal of the manuscript. TH performed the statistical analysis and helped to draft the manuscript. All authors read and approved the final manuscript.
